# Quantum correlation of microwave two-mode squeezed state generated by nonlinearity of InP HEMT

**DOI:** 10.1038/s41598-023-37739-0

**Published:** 2023-07-17

**Authors:** A. Salmanogli

**Affiliations:** Engineering Faculty, Electrical and Electronic Department, Cankaya University, Ankara, Turkey

**Keywords:** Electrical and electronic engineering, Quantum information, Quantum mechanics

## Abstract

This study significantly concentrates on cryogenic InP HEMT high-frequency circuit analysis using quantum theory to find how the transistor nonlinearity can affect the quantum correlation of the modes generated. Firstly, the total Hamiltonian of the circuit is derived, and the dynamic equation of the motion contributed is examined using the Heisenberg-Langevin equation. Using the nonlinear Hamiltonian, some components are attached to the intrinsic internal circuit of InP HEMT to address the circuit characteristics fully. The components attached are arisen due to the nonlinearity effects. As a result, the theoretical calculations show that the states generated in the circuit are mixed, and no pure state is produced. Accordingly, the modified circuit generates the two-mode squeezed thermal state, which means one can focus on calculating the Gaussian quantum discord to evaluate quantum correlation. It is also found that the nonlinearity factors (addressed as the nonlinear components in the circuit) can intensely influence the squeezed thermal state by which the quantum discord is changed. Finally, as the primary point, it is concluded that although it is possible to enhance the quantum correlation between modes by engineering the nonlinear components; however, attaining quantum discord greater than unity, entangled microwave photons, seems a challenging task since InP HEMT operates at 4.2 K.

## Introduction

Quantum correlation as a fundamental issue is significantly represented in quantum applications such as quantum information^1–7^ and quantum sensors^8–12^. The entanglement has been synonymously applied in the same way with quantum correlation in most applications since the quantum system contains only pure states^5^. In contrast, when the mixed states are generated by a quantum system, which covers most quantum systems, such as quantum radars^8,10^, the term entanglement cannot be used over quantum correlation. It is because some separable mixed states can introduce “residual correlation” that cannot be fitted by any classical probability distributions^5–7^. In other words, for a bipartite system with separable state ρ_AB_ expressed as ρ_AB_ = ∑P_i_ ρ_Ai_ × ρ_Bi_, where ρ_Ai_ and ρ_Bi_ are density matrices of the subsystems; the states ρ_Ai_ and ρ_Bi_ may be physically non-distinguishable. Consequently, all information about the subsystems cannot be locally retrieved due to the nonorthogonality of the states^7^. Some recently published studies have shown that classically correlated states might show the signature of quantumness ^13,14^. To evaluate the quantumness, the term “quantum discord” has been applied^1–7^. The quantum discord can quantify the residual correlation or the signature of quantumness^5–7^, which captures all quantum correlations in a bipartite state. However, several different quantifiers have been introduced to evaluate the nonclassicality of the correlation (quantum correlation); but the most popular one is quantum discord^15^. Quantum discord is the difference between the quantum correlation within a quantum state and its classical correlations^5–7^. Some studies show that quantum discord can be defied for both qubits and continuous variable systems^7,16^. Therefore, studying the quantum discord for the continuous variables is valuable due to the critical applications of the continuous variables, such as in quantum computation and quantum communications^17^. For some reason mentioned above, and since the circuit designed in this work interacts with the environment as any real quantum systems interact inevitably with the surrounding, we have to focus on the continuous variable and calculate the quantum discord for these variables. As the main point, the influence of the thermal noise generated by the circuit is studied on the quantum correlation. Thus, concerning the mentioned points, the study significantly focuses on the “Gaussian quantum discord” and uses the close formula satisfied to all families of the Gaussian state. Accordingly, the Gaussian state includes the important class of the squeezed thermal state. This unique state is realized by applying two-mode squeezing to a pair of single-mode thermal states^5,7^. Recent studies have shown that the squeezed thermal state, generally the Gaussian state, can be decomposed into the EPR (Einstien-Podolsky-Rosen) state plus the local action of a “phase-sensitive Gaussian channel”^5^.

As mentioned, this study significantly focuses on the two-mode squeezing thermally state, which is generated by the nonlinearity of the InP HEMT. In general, Indium Phosphide High Electron Mobility Transistor (InP HEMT) is known for its excellent high-frequency performance, low noise characteristics, and suitability for cryogenic applications. These properties make them attractive for use in quantum circuits and quantum information processing systems. The nonlinearity of the InP HEMT may play a crucial role in generating and manipulating quantum states and correlations within the circuit. In addition, the nonlinearity in an InP HEMT primarily arises due to the electron–electron interaction and the non-linear current–voltage characteristics of the device. The channel of the InP HEMT consists of a 2D electron gas, and the electrons interact with each other, leading to nonlinearity in the device’s behavior^16,18,19^.

It has to be initially shown that due to the nonlinearity of InP HEMT in the circuit, the states generated in the system are mixed. This means that the quantum discord as a quantifier can completely define the quantum correlation rather than the entanglement. Then, it will be verified that the introduced nonlinearity in the circuit generates the microwave’s two-mode squeezed thermal state^20^. Finally, it introduces some critical factors (components) relating to nonlinearity to enhance the quantum correlation between modes. The mentioned components, as the circuit elements, are attached to the original internal circuit of InP HEMT to make the modified circuit; Then, the modified circuit is simulated using PySpice. The simulation in PySpice is performed to check some critical issues from a circuit analysis point of view. Consequently, it will show that the attached nonlinearity can add a challenging trade-off.

It is better to indicate that this study completes the latter similar work^21^, in which, for simplicity, it was supposed that all states produced by the InP HEMT are pure states. Another difference between the present work with^21^ is that this study concentrates on the microwave two-mode squeezed thermal state and the squeezing parameters that affect the quantum correlation. Additionally, in this study, the thermally excited photons in the Drain due to the related conductance are introduced at 450 K (Drain conductance noise temperature), making the circuit behave like an accurate model.

The present study initially defines the system and its crucial elements that can affect the primary goal. In the next step, the system's Hamiltonian is derived and the dynamics equation of the motion is introduced using the nonlinear part of the Hamiltonian. In addition, it is shown that the circuit can generate a microwave two-mode squeezed thermal state by applying some nonlinear coefficients. In the other step, it is theoretically shown that the introduced circuit generates the mixed states. Finally, the theory related to quantum discord and its deriving is introduced and discusses by which parameters or factors one can manipulate the quantum discord.

## Theory and background

### System definition

**Figure 1:**
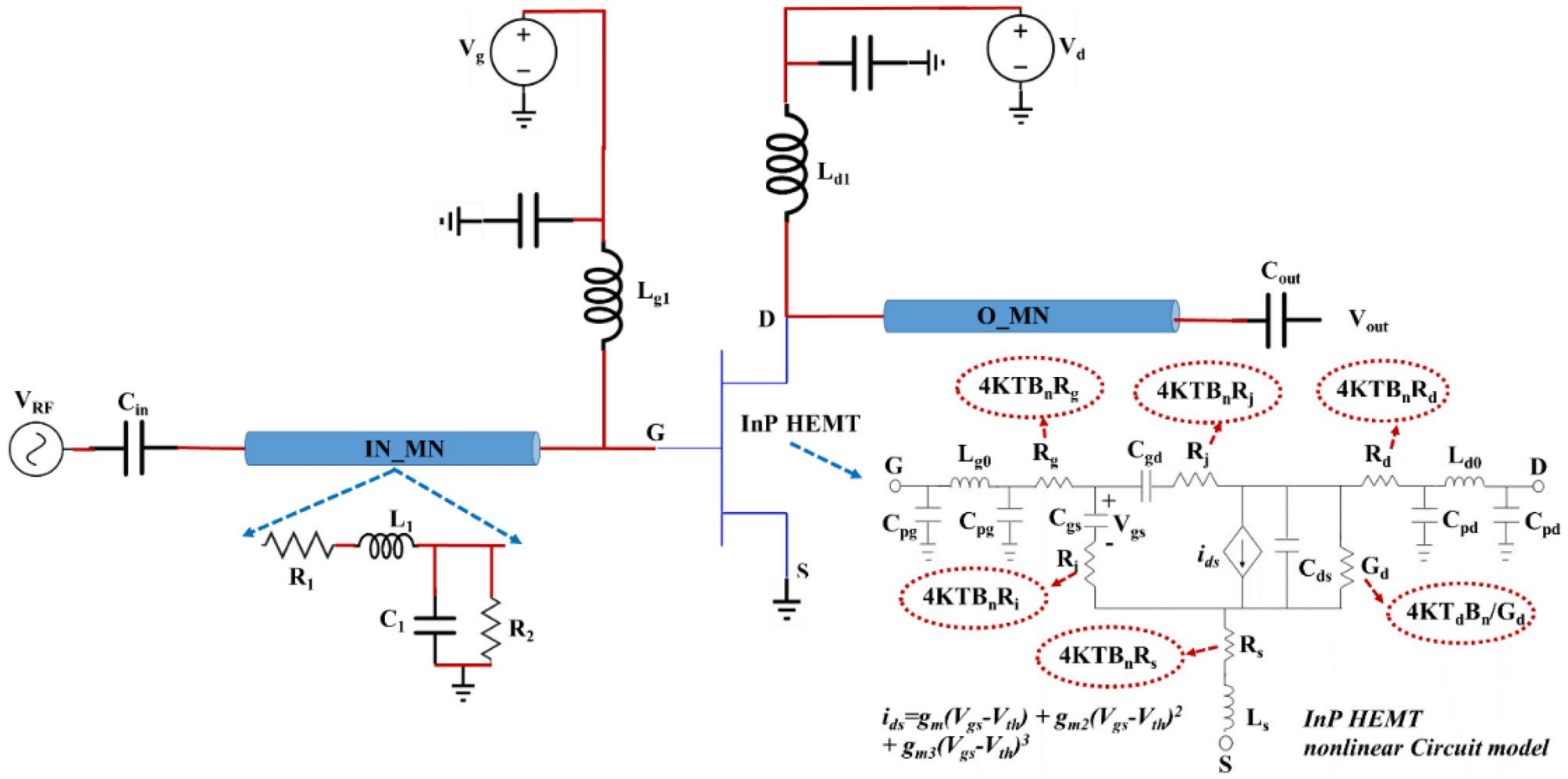
The schematic of the circuit containing the input and output matching networks, stability network, and active elements (InP HEMT) and its internal circuit.

The circuit studied in this work is schematically shown in Fig. 1. It contains InP HEMT as a nonlinear active element, an input and output matching network to match the input and output impedance, and a DC stabilization circuit. The generic equivalent circuits of the input and output matching network are shown in the inset figures. In the inset figures, it is shown in a usual way that any transmission line can be modeled with an equivalent lumped element circuit. As shown in the schematic, InP HEMT is biased via V_g_ and V_d_ to operate in the desired region, and the small signal RF input wave is applied to the circuit through an input capacitor (C_in_). L_g1_ and L_d1_ are key elements in the stabilization circuit. As an essential element, InP HEMT’s nonlinear equivalent circuit is attached as another inset figure. The attached inset tries to completely show all factors that could affect the operation of InP HEMT at cryogenic temperature; it is mainly for quantum applications in which the number of microwave photons is dramatically kept at a low level. For the reason that the thermally excited photons can affect the low photons quantum applications, the noise generated by resistances in the circuit is considered. This makes the circuit behave like a real one. For instance, 4KTB_n_R_s_ is the voltage-noise generated by R_s_ in the circuit, in which K, T, and B_n_ are, respectively, Boltzmann constant, operational temperature, and noise bandwidth.

One of the essential components in the inset circuit is i_ds,_ defined as the dependent current source controlled by the voltage. As can be seen from the related relationship, the amount of the current is controlled by g_m,_ defined as the intrinsic transconductance of the circuit; that is additionally manipulated by g_m2_ and g_m3_ called the second and third nonlinearity factors (generally called nonlinearity factors). This paper focuses on the latter factors and their effect on the circuit, through which the circuit’s resonance frequency is changed. In addition, the coupling signals between the resonators in the circuit can be strongly changed. The term “coupling between the resonators” is used because, from a general view, the circuit illustrated in Fig. 1 can be supposed to be the two separate oscillators oscillating in the gate and drain side of the transistor are coupled to each other through the InP HEMT nonlinear circuit^20^. In other words, the nonlinearity in InP HEMT changes the features of the oscillators, including the resonance frequency and their impedance.

### Hamiltonian of the circuit

**Figure 2:**
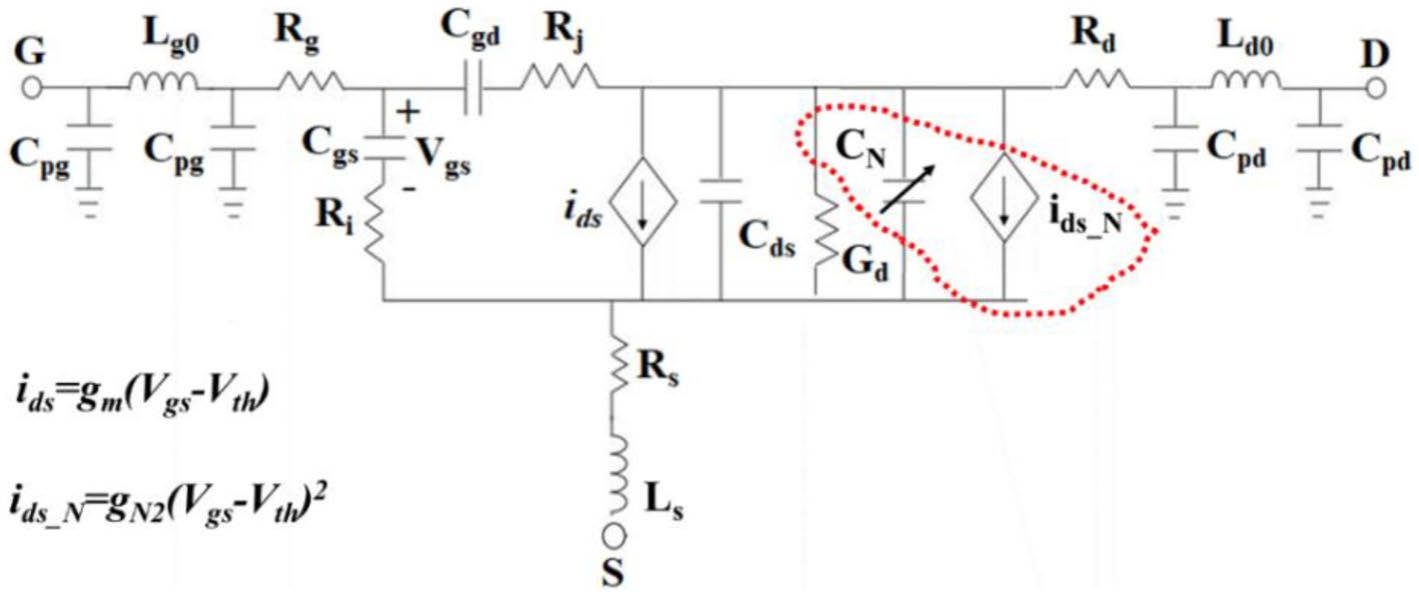
An approximation of the InP HEMT internal circuit by considering the nonlinearity effects illustration with a scribble-dotted line on the primary circuit.

To derive the classical Hamiltonian, one has to use Legendre transformation as H(φ_k_,Q_k_) = ∑_k_ (φ_k_.Q_k_)–L_c_, where L_c_ and Q_k_ are, respectively, the system’s Lagrangian and conjugate variables of the “coordinate variable” φ_k_, calculated through Q_k_ = ∂L_c_/∂(∂φ_k_/∂t)^21,22^. The total Hamiltonian of the circuit introduced in Fig. 1 is given by:

1 $$\begin{gathered} H_{t} = \frac{{C_{A} }}{2}\dot{\varphi }_{1}^{2} + \frac{1}{{2L_{1} }}\varphi_{1}^{2} + \frac{{C_{B} }}{2}\dot{\varphi }_{2} + \frac{1}{{2L_{2} }}\varphi_{2}^{2} - C_{c} \dot{\varphi }_{1} \dot{\varphi }_{2} \hfill \\ \,\,\,\,\,\,\,\,\,\,\,\, + \left\{ {g_{m2} \varphi_{2} \dot{\varphi }_{1}^{2} + 2g_{m3} \varphi_{2} \dot{\varphi }_{1}^{3} } \right\} - \varphi_{1} \left( {\overline{{I_{g}^{2} }} - \overline{{I_{i}^{2} }} } \right) \hfill \\ \,\,\,\,\,\,\,\,\,\,\, - \varphi_{2} \left( {\overline{{I_{ds}^{2} }} + \overline{{I_{d}^{2} }} + \overline{{I_{j}^{2} }} } \right) - \frac{{C_{in} }}{2}V_{rf}^{2} - \frac{{C_{gs} }}{2}\overline{{V_{i}^{2} }} , \hfill \\ \end{gathered}$$

where C_A_ = C_in_ + C_1_ + C_gs_ + C_gd_, C_B_ = C_gd_ + C_2_, and C_c_ = C_gd_. The total Hamiltonian of the system is written as H_t_ = H_L_ + H_N_, where H_L_ stands for linear Hamiltonian and H_N_ contains the nonlinear terms. The nonlinearity is arisen due to the terms inside the curly bracket. This study looks to generate two-mode squeezed thermal states using the nonlinearity introduced by InP HEMT. For this reason, the focus is just laid on H_N_ and its analysis, using which I. it will theoretically show that the generated states by the circuit presented in Fig. 1 are mixed, and II. there is the nonlinearity effect that can generate the two-mode squeezed thermal state. In contrast, H_L_ is used to define the steady-state operational point and energy level. The linear part of Hamiltonian has some terms that can generate the coherent state. The definition of H_L_ and its parameters have been introduced in Appendix A (Eqs. A1 and A2). Consequently, the nonlinear Hamiltonian is given by:

2 $$\begin{aligned} H_{N} & = \frac{{g_{N}^{2} }}{{C_{M}^{4} }}\left\{ {C_{B}^{2} \varphi_{2} Q_{1}^{2} + C_{c}^{2} \varphi_{2} Q_{2}^{2} + g_{m}^{2} C_{B}^{2} \varphi_{2}^{3} } \right. \\ & \quad - \left. { 2g_{m} C_{B}^{2} Q_{1} \varphi_{2}^{2} - 2g_{m} C_{B} C_{c} Q_{2} \varphi_{2}^{2} + 2g_{m} C_{B} C_{c} Q_{1} \varphi_{1} \varphi_{2} } \right\}, \\ \end{aligned}$$

where g_N2_ = g_m2_ + 6g_m3_[∂φ_1_/∂t]_DC_, C_M_^2^ = C_B_(C_A_ + C_N_)-C_c_^2^, and C_N_ = g_m2_[φ_2_]_DC_ + 6g_m3_[φ_2_]_DC_*[∂φ_1_/∂t]_DC_. In the defined relationships, g_N2_ and C_N_ are estimated using the approximation methods and are defined as the nonlinearity factor and nonlinear capacitance. It is clearly shown in Eq. (2) that the g_N2_ and C_N_ strongly manipulate the amplitude of the nonlinearity created in InP HEMT. Additionally, it is shown that the resonance frequency of the second oscillator is dramatically dependent on C_N_. This means that the nonlinearity in InP HEMT can affect the resonance frequency, which is calculated as ω_2_ = 1/√(C_q2_L_2’_); in this equation, C_q2_ and L_2’_ expressed in detail in Appendix A (Eq. A_2_) are affected by the nonlinearity factors. To clarify the effects of the nonlinearity, some components created because of the InP HEMT nonlinearity are attached to the main circuit (inset figure in Fig. 1) and illustrated in Fig. 2. These elements are theoretically derived using nonlinear Hamiltonian expressed in Eq. (2). The red scribble-dotted line on the circuit contains a variable capacitor (C_N_) and controllable current depending on the nonlinearity factors of the InP HEMT.

The circuit illustrated in Fig. 2 is an approximated circuit that may model the InP HEMT transistor behavior. The present study uses this model to quantum mechanically analyze the InP HEMT nonlinearity effect on the quantum correlation that may occur between modes generated in the circuit at cryogenic temperature. As clearly seen in Fig. 2, C_N_ and i_ds_N_ directly affect the second oscillator resonance properties; nonetheless, the nonlinear effects are coupled to the first oscillator through C_gd_. This means that the coupling properties of the oscillators are strongly affected by the nonlinear properties. For example, the cross-correlation between the coupled oscillator’s modes is severely influenced by the nonlinearity factors defined in the circuit. In other words, the classicality correlated modes generated by the coupled oscillators can be affected in such a way as to show quantumness. This phenomenon strongly depends on nonlinearity and its impacts. As an important quantifier, the quantum discord^5–7^ is selected to evaluate the quantum correlation between the states generated in the circuit. The quantum discord rather than the entanglement is chosen to show the effects because the circuit discussed in this study shows mixed states. This point will be explored in the next section. In the complementary part of this study, the circuit shown in Fig. 2 is analyzed using PySpice to investigate the effect of the attached components on InP HEMT’s main feature, such as DC characterization. Using PySpice, rather than any specific CAD simulator tools, gives some degree of freedom by which the designer can easily attach any elements or sub-elements to the circuit and make the desired pack. We also selected PySpice to work with because all theoretical simulations to calculate the mean photons number, quantum discord, the smaller Symplectic eigenvalue, and quantum mutual information were done in Python.

It should be noted that the general aim of the attached elements to the circuit is to study their effects on the performance of InP HEMT operating at 4.2 K and determine by what factors it can manipulate the quantum correlation between modes generated in the circuit. In the following, it is necessary to re-express Eq. (2) in the form of the ladder operator to analyze the quantum correlation between the modes generated by the circuit’s nonlinearity. Using the traditional methods, the nonlinear Hamiltonian in terms of the ladder operator is expressed as:

3 $$\begin{aligned} H_{N} &= \frac{{g_{N2} }}{{C_{M}^{4} }}\left\{ { - C_{B}^{2} \sqrt {\frac{{\hbar Z_{2} }}{2}} \frac{\hbar }{{2Z_{1} }}\left( {a_{2} + a_{2}^{ + } } \right)\left( {a_{1} - a_{1}^{ + } } \right)^{2} - C_{c}^{2} \sqrt {\frac{{\hbar Z_{2} }}{2}} \frac{\hbar }{{2Z_{2} }}\left( {a_{2} + a_{2}^{ + } } \right)\left( {a_{2} - a_{2}^{ + } } \right)^{2} } \right.\, + g_{m}^{2} C_{B}^{2} \sqrt {\frac{{\hbar Z_{2} }}{2}} \frac{\hbar }{{2Z_{2} }}\left( {a_{2} + a_{2}^{ + } } \right)^{3} \\ & + i2g_{m} C_{B}^{2} \sqrt {\frac{\hbar }{{2Z_{1} }}} \frac{{\hbar Z_{2} }}{2}\left( {a_{1} - a_{1}^{ + } } \right)\left( {a_{2} + a_{2}^{ + } } \right)^{2} + i2g_{m} C_{B} C_{c} \sqrt {\frac{\hbar }{{2Z_{2} }}} \frac{{\hbar Z_{2} }}{2}\left( {a_{2} - a_{2}^{ + } } \right)\left( {a_{2} + a_{2}^{ + } } \right)^{2} - \left. {i2g_{m} C_{B} C_{c} \sqrt {\frac{\hbar }{{2Z_{1} }}} \frac{{\hbar \sqrt {Z_{1} Z_{2} } }}{2}\left( {a_{1} - a_{1}^{ + } } \right)\left( {a_{1} + a_{1}^{ + } } \right)\left( {a_{2} + a_{2}^{ + } } \right)} \right\}, \\ \end{aligned}$$

where a_k_ and a_k_^+^ (k = 1, 2) are the annihilation and creation operators, respectively. For simplicity, a few constants are defined by which Eq. (3) is simplified as:

4 $$\begin{gathered} H_{N} = \left\{ { - \hbar g_{N11} \left( {a_{2} + a_{2}^{ + } } \right)\left( {a_{1} - a_{1}^{ + } } \right)^{2} - \hbar g_{N21} \left( {a_{2} + a_{2}^{ + } } \right)\left( {a_{2} - a_{2}^{ + } } \right)^{2} } \right. + \hbar g_{N31} \left( {a_{2} + a_{2}^{ + } } \right)^{3} \hfill \\ \,\,\,\,\,\,\,\,\, + i\hbar g_{N41} \left( {a_{1} - a_{1}^{ + } } \right)\left( {a_{2} + a_{2}^{ + } } \right)^{2} + i\hbar g_{N51} \left( {a_{2} - a_{2}^{ + } } \right)\left( {a_{2} + a_{2}^{ + } } \right)^{2} - \left. {i\hbar g_{N61} \left( {a_{1} - a_{1}^{ + } } \right)\left( {a_{1} + a_{1}^{ + } } \right)\left( {a_{2} + a_{2}^{ + } } \right)} \right\}, \hfill \\ \end{gathered}$$

where g_N11_, g_N21_, g_N31_, g_N41_, g_N51_, and g_N61_, are the related constants defined in Appendix A (Eq. A3). These constants are strongly dependent on the nonlinearity factor. The dynamics equation of motion of the circuit is calculated using the Heisenberg-Langevin equation; A simple way to achieve a stationary and robust calculation in continuous modes to calculate quantum correlation, i.e. entanglement^23,24^ or quantum discord^9^, is to select a constant point that the system is driven and works with. Since the interaction field is so strong, it is appropriate to focus on linearization and calculate the quantum fluctuation around the semi-classical constant point. For linearization, the oscillator modes are expressed as the stationary (constant) summation and fluctuating parts as **a**_**1**_ = A_1_ + **δa**_**1**_ and **a**_**2**_ = A_2_ + **δa**_**2**_, where the capital letter (A_1_ and A_2_) denotes the system’s steady-state points, and **δ** indicates the fluctuation around the steady-state point. The steady-state points related to the circuit are calculated and presented in Appendix A (Eq. A_7_). Thus, the linearized equations around the steady-state points are given by:

5 $$\begin{gathered} \mathop {\delta a_{1} }\limits^{ \bullet } = - \left( {i\omega_{1} + \frac{{\kappa_{1} }}{2}} \right)\delta a_{1} + \gamma_{a11} \left( {\delta a_{1} + \delta a_{1}^{ + } } \right) + \gamma_{a12} \left( {\delta a_{2} + \delta a_{2}^{ + } } \right) + \gamma_{a13} \delta a_{1}^{ + } + \sqrt {2\kappa_{1} } \delta a_{in - 1} \hfill \\ \mathop {\delta a_{2} }\limits^{ \bullet } = - \left( {i\omega_{2} + \frac{{\kappa_{2} }}{2}} \right)\delta a_{2} + \gamma_{a21} \left( {\delta a_{1} - \delta a_{1}^{ + } } \right) + \gamma_{a22} \left( {\delta a_{2} + \delta a_{2}^{ + } } \right) + \gamma_{a23} \left( {\delta a_{2} - \delta a_{2}^{ + } } \right) + \gamma_{a24} \left( {\delta a_{1} + \delta a_{1}^{ + } } \right) + \sqrt {2\kappa_{2} } \delta a_{in - 2} , \hfill \\ \end{gathered}$$

where γ_a11_, γ_a12_, γ_a13_, γ_a21_, γ_a22_, γ_a23_, and γ_a24_ are constant rates depending on the stationary points of the system. These rates can be complex numbers defined in Appendix A (Eq. A_4_). In Eq. (5), κ_1_, κ_2_, ω_1_, and ω_2_ are the first and second oscillators’ decay rates and the contributed frequencies, respectively. Additionally, δa_in_1_ and δa_in_2_ are the input noises fluctuation. They obey the correlation function < δa_in_1_(s)δa_in_1_^+^(s’) >  = [1 + N(ω)] × **δ**(s–s’), where N(ω) = [exp(ћω/k_B_T)-1]^-1^, in which k_B_ and T are the Boltzmann’s constant and operational temperature, respectively^9,21,22^. N(ω) is the equilibrium mean of the thermal photon numbers at the frequency ω. Finally, capital **δ** expressed in **δ**(s–s’) is Dirac’s function. In the following, Eq. (5) is transformed to the Fourier domain to simplify the algebra in the frequency domain to calculate the quantum discord, which is introduced as:

6 $$\begin{gathered} \left( {i\Delta_{1} + \frac{{\kappa_{1} }}{2} - \gamma_{a11} } \right)\delta a_{1} = \left( {\gamma_{a11} + \gamma_{a13} } \right)\delta a_{1}^{ + } + \gamma_{a12} \left( {\delta a_{2} + \delta a_{2}^{ + } } \right) + \sqrt {2\kappa_{1} } \delta a_{in - 1} \hfill \\ \left( {i\Delta_{2} + \frac{{\kappa_{2} }}{2} - \gamma_{a22} - \gamma_{a23} } \right)\delta a_{2} = \gamma_{a21} \left( {\delta a_{1} - \delta a_{1}^{ + } } \right) + \left( {\gamma_{a22} + \gamma_{a23} } \right)\delta a_{2}^{ + } + \gamma_{a24} \left( {\delta a_{1} + \delta a_{1}^{ + } } \right) + \sqrt {2\kappa_{2} } \delta a_{in - 2} , \hfill \\ \end{gathered}$$

where ∆_1_ and ∆_2_ are the oscillators detuning frequencies, which are calculated as ∆_1_ = ω–ω_1_ and ∆_2_ = ω–ω_2_, where ω is the RF incident frequency. Notably, Eq. (6) is used to calculate the < δa_1_^+^δa_1_ > , < δa_2_^+^δa_2_ > , and < δa_1_δa_2_ > as the mean photon number of the first-, second-oscillator, and the phase-sensitive cross-correlation^17,21,22,25^.

### Mixed states generation because of the nonlinearity of InP HEMT

This part shows that the state of the oscillators is dispersed, meaning that all states generated by the circuit become mixed states. One can utilize the first perturbation theory to calculate the oscillator’s state changes. The change of a typical state is calculated using the first perturbation theory by |j > ^(1)^ = ∑_i≠j_ {(< i|H_N_|j >)/(E_i_-E_j_)} ×|j > ^22^, where |j > is the pure state of the oscillators, and E_i_ and E_j_ are the contributed energies. |j > ^(1)^ is the final state of the first oscillator that may differ from |j > ; it depends on the system and the related Hamiltonian. The results of the calculation are presented in Eq. (7) as:

7 $$\begin{gathered} \left| {j_{1} } \right\rangle^{(1)} = \sum\limits_{{i \ne j_{1} }} {\frac{{\left\langle {i|H_{N} |j_{1} } \right\rangle }}{{E_{i} - E_{j1} }}} \left| {j_{1} } \right\rangle = \frac{{j_{p11} }}{{E_{{(j_{1} - 2)}} - E_{j1} }}\left| {j_{1} - 2} \right\rangle + \frac{{j_{p12} }}{{E_{{(j_{1} + 2)}} - E_{j1} }}\left| {j_{1} + 2} \right\rangle + \frac{{j_{p13} }}{{E_{{(j_{1} - 1)}} - E_{j1} }}\left| {j_{1} - 1} \right\rangle - \frac{{j_{p13} }}{{E_{{(j_{1} + 1)}} - E_{j1} }}\left| {j_{1} + 1} \right\rangle \hfill \\ \hfill \\ \left| {j_{2} } \right\rangle^{(1)} = \sum\limits_{{i \ne j_{1} }} {\frac{{\left\langle {i|H_{N} |j_{2} } \right\rangle }}{{E_{i} - E_{j2} }}} \left| {j_{2} } \right\rangle \, = \frac{{j_{p21} }}{{E_{{(j_{2} - 3)}} - E_{j2} }}\left| {j_{2} - 3} \right\rangle + \frac{{j_{p21} }}{{E_{{(j_{2} + 3)}} - E_{j2} }}\left| {j_{2} + 3} \right\rangle + \frac{{\left[ {j_{p23} + j_{p27} + i\left( {j_{p26} + j_{p27} + j_{p28} } \right)} \right]}}{{E_{{(j_{2} - 1)}} - E_{j2} }}\left| {j_{2} - 1} \right\rangle \hfill \\ \hfill \\ \,\,\,\,\,\,\,\,\,\,\,\,\,\,\,\,\,\,\,\,\,\,\,\,\,\,\,\,\,\,\,\,\,\,\,\,\,\,\,\,\,\,\,\,\,\,\,\,\,\,\,\,\,\,\,\,\,\,\, + \frac{{\left[ {j_{p24} + j_{p25} + i\left( {j_{p25} + j_{p29} + j_{p210} } \right)} \right]}}{{E_{{(j_{2} + 1)}} - E_{j2} }}\left| {j_{2} + 1} \right\rangle + \frac{{j_{p211} }}{{E_{{(j_{2} + 2)}} - E_{j2} }}\left| {j_{2} + 2} \right\rangle + \frac{{j_{p212} }}{{E_{{(j_{2} - 2)}} - E_{j2} }}\left| {j_{2} - 2} \right\rangle . \hfill \\ \end{gathered}$$

In this equation, the data related to the constants used are given in Appendix A (A6). This equation shows that the final states related to the first and second oscillators |j_1_ > ^(1)^ and |j_2_ > ^(1)^, are strongly influenced by the nonlinear (perturbation) Hamiltonian. For example, the state of the first oscillator is coupled to |j_1_ ± 1 > and | j ± 2 > due to the nonlinearity effect. For instance, if one fixed the first oscillator state at |0 > as a pure state, the final state of this oscillator will be found in the superposition state of |1 > and |2 > , meaning that H_N_ causes the final states to be mixed. In other words, the state |0 > is mixed with |1 > and |2 > .

In addition, it is necessary to calculate the energy of the oscillators using E_j_ =  < j_i_|H_0_ + H_N_|j_i_ > ^22^, where j_i_ = 1,2. The oscillators associated energies due to the total Hamiltonian are given by:

8 $$\begin{gathered} E_{{j_{1} }} = \left\langle {j_{1} |H_{0} + H_{N} |j_{1} } \right\rangle = \hbar \omega_{1} \left( {j_{1} + \frac{1}{2}} \right) + \hbar \left\{ {2g_{N11} {\text{Re}} (A_{2} ) - i2g_{N61} {\text{Re}} (A_{2} )} \right\} \hfill \\ E_{{j_{2} }} = \left\langle {j_{2} |H_{0} + H_{N} |j_{2} } \right\rangle \, = \hbar \omega_{2} \left( {j_{2} + \frac{1}{2}} \right) + i\hbar \left\{ {2g_{N41} (2j_{2} + 1)} \right\}. \hfill \\ \end{gathered}$$

From Eq. (8), it is clear that the first term is arisen due to the linear Hamiltonian, and the terms inside the curly bracket are generated because of the nonlinear Hamiltonian effect. It is called the perturbation effect on the energy levels of the oscillators. In this equation, A_1_ and A_2_ are the steady-state points (DC points) of LC_1_ and LC_2_, where the oscillators are designed to operate on. The DC points can be calculated using Heisenberg-Langevin equations in the steady-state^15,16^; these points are essentially affected by the circuit’s DC bias and the linear Hamiltonian effect. The DC points related to the circuit are calculated and presented in Appendix A (Eq. A7). The equation shows that the circuit's steady state points strongly depend on the circuit's generated noise (shown in Fig. 1), and the DC bias point. As an essential result of this research, it is found that the quantities such as g_12_^’^, g_22_^’^, and C_q1q2_ handling the displacement in the circuit (linear Hamiltonian) strongly affect the steady-state points. This gives any engineer a degree of freedom to control and manipulate the steady-state point in which the circuit is established to be operated.

### Two-mode squeezed thermal state

A squeezed-coherent state is created via the acting of the squeezed and displacement operators on the vacuum state defined as |α,ζ >  = D(α)S(ζ)|0 > , where |0 > is the vacuum state^20,22^. In this study, it can be easily shown that the linear part of the total Hamiltonian can generate a coherent state. In contrast, a squeezed state for each oscillator needs quadratic terms such as a_i_^2^ and a_i_^+2^ in the exponent. Nonetheless, for two-mode squeezing, the Hamiltonian should have the terms like {a_1_a_2_ + a_1_^+^a_2_^+^}^20,22,26–29^. Therefore, the two-mode squeezed state becomes analyzed by the evolution of exp[H_N_t/iħ], where H_N_ is defined in Eq. (4). Based on this definition, any quadratic terms like {a_1_a_2_ + a_1_^+^a_2_^+^} in the Hamiltonian may generate two-mode squeezing. Consequently, using exp[H_N_t/iħ], the two-mode squeezing state of the nonlinear circuit studied is presented by:

9 $$\begin{gathered} S\left( {\zeta_{12} } \right) = \exp \left[ { - \hbar g_{N11} \left( {a_{2} + a_{2}^{ + } } \right)\left( {a_{1} - a_{1}^{ + } } \right)^{2} } \right. + i\hbar g_{N41} \left( {a_{1} - a_{1}^{ + } } \right)\left( {a_{2} + a_{2}^{ + } } \right)^{2} \, - i\hbar g_{N61} \left( {a_{1} - a_{1}^{ + } } \right)\left( {a_{1} + a_{1}^{ + } } \right)\left( {a_{2} + a_{2}^{ + } } \right)\left. {} \right]\frac{t}{i\hbar } \hfill \\ \hfill \\ S\left( {\zeta_{12} } \right) = \exp \zeta_{12} \left( {a_{2} a_{1} - a_{2}^{ + } a_{1}^{ + } } \right)t. \hfill \\ \end{gathered}$$

Equation (9) shows that the coupled oscillator can generate two-mode squeezing through the transistor’s nonlinearity. That means that the nonlinearity created by the transistor couples two oscillators so that the coupled two-mode become squeezed. The two-mode squeezing parameter is defined as ζ_12_ = − 2g_N11_Im(A_1_) + 2g_N41_Re(A_2_) + 2g_N61_Re(A_1_), where Re{} and Im{} indicate the real and imaginary parts, respectively. It is apparent from the relationship that ζ_12_ can be a complex number, which means that the two-mode squeezing parameter contains a phase, which determines the angle of the quadrature.

It should be noted that “*t*” in the exponent (exp[Ht/jħ]) can be determined from *t* < min{1/κ_1_, 1/κ_2_}, where κ_1 _and κ_2 _are the first and second oscillators’ decay rates. By selecting “*t*”, the system is forced to generate squeezing before the resonator decaying^20^.

### Quantum discord

The calculation of the quantum discord initiates using two generalizations of the classical mutual information^1–7^. The mutual information is used primarily to evaluate the total correlations between subsystems^15^. In the first generalization, the quantum mutual information for two systems, A and B, is defined as I(ρ_AB_) = S(ρ_A_) + S(ρ_B_)–S(ρ_AB_), where S(ρ_A_) = − Tr(ρ_A_log_2_ρ_A_) is the von Neumann entropy of system A, and S(ρ_AB_) is the conditional von Neumann entropy^5–7^. In the following, for the system studied in this work, the first oscillator is shortly called A, and the second one is called B. The conditional entropy arises because the measurement process disturbs the state on which a physical system is set. In other words, the applied measurement on subsystem B may change the state of subsystem A.

The second generalization introduces the entropic quantity C(A|B), by which the classical correlation in the joint state ρ_AB_ is calculated. The classical correlation defines the maximum information about one subsystem depending on the measurement types applied to the other subsystem. The entropic quantity, by considering the generalization, is defined as C(ρ_AB_) = S(ρ_A_)–S_min_(ρ_AB_), where the only difference between mutual quantum information is S_min_(ρ_AB_). This term is the conditional minimized entropy of system A over all possible measurements on system B. That is generally described as positive operator valued measures (POVMs)^5–7^. Thus, quantum discord is usually defined as D(ρ_AB_) = I(ρ_AB_)–C(ρ_AB_), and by substituting from the above definitions, it becomes D(ρ_AB_) = S(ρ_B_)–S(ρ_AB_) + S_min_(A|B).

Fortunately, a compact formula can be presented for two-mode squeezed thermal states (zero-mean Gaussian states) to reduce the covariance matrix (CM) into the standard form^5^. Consequently, the CM of the selected modes in the system can be presented in the form of the following matrix:

10 $$V_{AB} = \left( {\begin{array}{*{20}c} {(\tau b + \eta )I} & {} & {\sqrt {\tau \left( {b^{2} - 1} \right)} C} \\ {} & {} & {} \\ {\sqrt {\tau \left( {b^{2} - 1} \right)} C} & {} & {bI} \\ \end{array} } \right),$$

where **I** ≡ diag(1,1), **C** ≡ diag(1,− 1), a = n_o1_ + 0.5, b = n_o2_ + 0.5, τ = d_o12_^2^/(b^2^–1), and η = a–(b_2_* d_o12_^2^/(b^2^–1))^9^. In these equations, a and b are the expectation value of the I/Q signals for two oscillators derived as a ≡ < I_1_(ω)I_1_(ω) >  =  < Q_1_(ω)Q_1_(ω) > , b ≡ < I_2_(ω)I_2_(ω) >  =  < Q_2_(ω)Q_2_(ω) > , where I = (δa_j_^+^  + δa_j_)/√2 and Q = (δa_j_—δa_j_^+^)/i√2, for subscript j = 1,2. In the listed relationships, n_o1_, n_o2_, and d_o12_ are the output mean photon numbers of the first and second oscillator and the cross-correlation phase-sensitive, respectively. One can use the input–output formula^22^ to calculate the output mean photon numbers in the form of n_o1_ = 2κ_1_ < δa_1_^+^δa_1_ >  +  < δa_in-1_^+^δa_in-1_ > , n_o2_ = 2κ_2_ < δa_2_^+^δa_2_ >  +  < δa_in-2_^+^δa_in-2_ > , d_o12_ = 2√(κ_1_κ_2_) < δa_1_δa_2_ > . Finally, using Eq. (6), the mean photon numbers of the oscillators are calculated as n_1_ ≡ < δa_1_^+^δa_1_ > , n_2_ ≡ < δa_2_^+^δa_2_ > , and d_12_ ≡ < δa_1_δa_2_ >^9,21^, where n_1_, n_2_, and d_12_ are, respectively, the first-, second oscillator mean photon number, the cross-correlation between two mentioned oscillators. It is supposed that d_12_ is real-valued.

The output entropy associated with the heterodyne detection is equal to the average entropy of the output ensemble A^5^. Since entropy is invariant under displacements, it may write S(A|het_B_) = h(τ + η), where h(x) ≡ (x + 0.5)log_2_(x + 0.5)–(x–0.5)log_2_(x–0.5). In other words, the von Neumann entropy of an n-mode Gaussian state with CM expressed in Eq. (10) is calculated as S(V_AB_) = ∑_i=1_^N^ f(ν_i_), where ν_i_ are the related Symplectic eigenvalues^6^. However, there is a heterodyne detection for which it is optimal for minimization of the output entropy, so the Gaussian discord is optimal. The “Gaussian quantum discord” of a two-mode Gaussian state ρ_AB_, assuming a two-mode squeezed thermal state in this study, can be defined as the quantum discord satisfying the conditional entropy restricted to the generalized Gaussian POVMs on B^6^.

Finally, the compact form of quantum discord, classical correlation, and quantum mutual information are given, respectively, by D(ρ_AB_) = h(b)–h (ν_-_)–h (ν_+_) + h (τ + η), C(ρ_AB_) = h(a)–h (τ + η), and I(ρ_AB_) = h(a)–h (ν_-_)–h (ν_+_), where ν_±_ is the Symplectic eigenvalue of the CM. The Symplectic eigenvalues are defined as ν_±_  = [∆ ± √(∆^2^-4D)]/2, where ∆ = det(a**I**) + det(b**I**) + 2det(d_o12_**I**) and D = det(V_AB_); in the recent formulas det{:} stands for the matrix determinant. In the compact form of the equation defined for the quantum discord, the first term stands for the von Neumann entropy of the second oscillator in the system. The second and third terms define the von Neumann conditional entropy of the system. The last term in the equation is the effect of the classical correlation depending on the type of measurement performed on the second oscillator. As a result, in the system defined, the second oscillator entropy and the off-diagonal elements in the CM significantly affect the system's quantum discord. For this reason, in this article, we specially pay attention to the interferences between the oscillators and determine which quantities can affect the quantum discord. Perhaps the other critical factor that may be considered to enhance the quantum discord is h(τ + η), by which the classical correlation is decreased. As mentioned in^5–7^, this critical factor strongly depends on the type of measurements.

### Ethical approval and consent to participate

This study involves no human participants, human data, or related data.

## Results and discussions

In the following, the quantum correlation generated between the two-mode squeezing state by the circuit shown in Fig. 1 is discussed. It attempted to focus on the crucial parameters in the circuit (Fig. 2) that one can manipulate in such a way as to enhance the quantum correlation between modes. As shown in Fig. 2, C_N_ and g_N2_ are two crucial factors, mainly the function of g_m2_ and g_m3_, that the study concentrates on them to manipulate the quantum correlation. Notably, it was theoretically shown in the latter section that g_N2_ is the main factor by which the final states of the oscillators became mixed and also plays a significant role in generating the two-mode squeezing thermal state.

Using the information mentioned above, in the following, we attempt to show that the quantum correlation can be generated between the microwave photons created by the two coupled oscillators. As a quantifier, the quantum discord is calculated, a property almost all quantum states hold. However, other quantities, such as the quantum information and classical correlation generated between modes in the system, are analyzed. The author thinks that comparing the quantum discord, quantum information, and classical correlation gives a solid sense for anyone to find which parameter can strongly enhance the quantum correlation. In addition, it makes it possible to know what portion of the classical correlation may appear in the quantum correlation. This means that all classically correlated states don’t retain signatures of quantumness.

Before discussing the simulation results, it is necessary to care about a more severe and crucial point, which is InP HEMT operating at 4.2 K; this makes an astonishing thermally exciting noise in the circuit. Also, the internal circuit effects cause the quantum discord amplitude to remain limited. Thus, it seems impossible to compare the results of this study with the quantum discord generated by a typical Opto-mechanical-microwave system’s quantum discord^9^. It should be noted that the recent system works at 10 mK, whereas InP HEMT operates at 4.2 K; this means that the thermally excited photons dramatically affect the quantum correlation.

In this study, one of the aims is to design a circuit with an operating frequency (f_inc_ = f_1_ + f_2_; f_1_ ~ 5.5 GHz, f_2_ ~ 6.5 GHz) of around 12 GHz, where f_inc_, f_1_, and f_2_ are the RF incident frequency, the first and second oscillator’s frequency, respectively. In the range of mentioned frequency and operating at 4.2 K, the mean thermally exciting photons were calculated as n_th1_ ≡ < δa_in_1_^+^δa_in_1_ > or n_th2_ ≡ < δa_in_2_^+^δa_in_2_ >  ~ 12, which is so greater than the average number of photons generated by the first and second oscillators n_1_ ≡ < δa_1_^+^δa_1_ > or n_2_ ≡ < δa_2_^+^δa_2_ >  ~ 0.2. With knowledge of the thermally excited photons as the inserting noise and also the oscillators’ mean photon number, in the following, the Gaussian quantum discord of the two-modes Gaussian states generated by the coupled oscillators is calculated. All simulations in this work containing the theoretical simulation of quantum correlation and also the simulation of the nonlinearity effects on the InP HEMT DC characteristics in PySpice were performed using the data from Table 1.

**Table 1:** Values for the small signal model of the 4×50 μm InP HEMT at 5 K^18,19^.

	Stands for	Value [unit]
R_g_	Gate resistance	0.3 Ω
L_g_	Gate inductance	75 pH
L_d_	Drain inductance	70 pH
C_gs_	Gate-source capacitance	107 fF
C_ds_	Drain-source capacitance	51 fF
C_gd_	Gate-drain capacitance	60 fF
R_i_	Gate-source resistance	0.07 Ω
R_j_	Gate-drain resistance	8 Ω
g_d_	Drain-source conductance	12 mS
g_m_	Intrinsic transconductance	82 mS
V_g_	Gate bias voltage	0.03 V
V_d_	Drain bias voltage	0.06 V
T	Operational temperature	4.2 K
T_d_	Drain noise temperature	450 K

**Figure 3:**
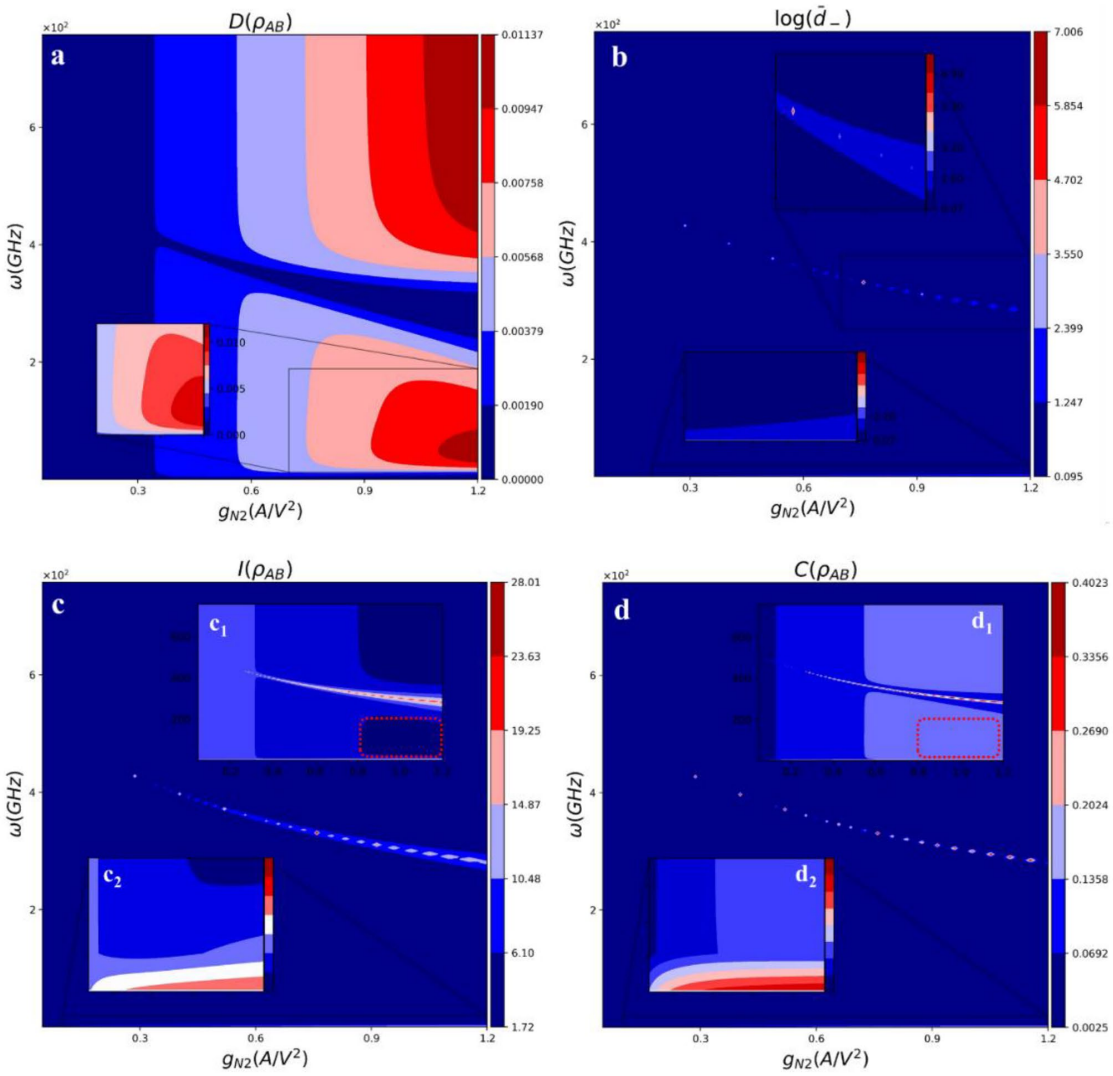
(**a**) Quantum discord, (**b**) smaller Symplectic eigenvalue (log_10_[đ_-_]), (**c**) Quantum mutual Information; c_1_ and c_2_: log_10_[I(ρ_AB_)], (**d**) classical correlation; d_1_ and d_2_: log_10_[C(ρ_AB_)], vs. angular frequency (GHz), and nonlinear factor g_N2_ (A/V^2^).

The simulation results for quantum discord, the smaller Symplectic eigenvalue, quantum mutual information, and classical correlation at 4.2 K are shown in Fig. 3. The result in Fig. 3a indicates that increasing nonlinearity factor, g_N2_, significantly enhances the quantum correlation. The graph shows that the quantum discord is strongly improved in the two different frequency ranges. It means that there is an avoided-level crossing in the graph (like a gap), where the quantum correlation between modes vanishes. According to the demonstrated figure, D(ρ_AB_) fluctuates around zero in some regions. It has been shown that a state with zero quantum discord represents a classical probability, while a positive discord indicates quantumness even in a separable mixed state^6,7^. Therefore, for 0 < D(ρ_AB_) < 1, the states may be entangled or separable, but for D(ρ_AB_) > 1, the states are entangled.

Generally, using the results illustrated, this paper wants to answer a few important questions: 1. Is it possible to generate quantum correlation using InP HEMT nonlinearity operating at 4.2 K? 2. By what factor the nonlinearity generated by the InP HEMT can affect the quantum correlation? 3. Is it possible to generate entangled microwave photons at 4.2 K? The Gaussian quantum A discord is calculated to answer the mentioned questions, and the measurement is performed on the second oscillator to minimize the classical correlation.

Figure 3a demonstrates that the amplitude of quantum A discord is less than unity 0 < D(ρ_AB_) < 1, which means that the states generated by the oscillators may have either separable or entangled states. Consequently, if one applies a real InP HEMT operating at 4.2 K and considers all the noises and nonlinearity effects (Fig. 2), the generation of the entangled microwave photons seems impossible. This is mainly because more thermally excited photons are generated in microwave frequencies. Nonetheless, it is possible to create a partial quantum correlation between the two-mode Gaussian states (mixed states in this study). An inset figure is attached to Fig. 3a to show the critical points where the quantum discord is maximized. However, the interesting fact about this graph is the gap at which the quantum discord is minimized or completely disappeared. To discuss this odd behavior, the smaller Symplectic eigenvalue (đ_-_) of the partially transposed state is illustrated in Fig. 3b. For better illustration, the logarithmic scale of the quantity (log_10_(đ_-_)) is shown, at which two inset figures are attached. The minimum value for đ_-_ is around 1.1 (log_10_(1.1) = 0.095). It has been shown that the Gaussian state is entangled if and only if đ_-_ < 0.5^7^ (đ_-_ < 1^6^). As a result, the curve illustrated in Fig. 3b shows that the state generated by the circuit is not entangled because d_-_ is always greater than 1.1 for each g_N2_. In addition, the study focuses on the smaller Symplectic eigenvalue because there is a pure consistency between the two figures illustrated in Fig. 3a and b. It can be found that the more quantum discord the circuit can generate, the less đ_-_ the circuit can produce.

However, we learned from the latter section that some other quantifiers could help to elucidate the quantum discord’s behavior. In other words, the difference between quantum discord, quantum mutual information, and classical correlation was mathematically discussed in the latter section. Thus, to thoroughly analyze the quantum discord, the simulation results of the quantum information and classical correlation are illustrated in Fig. 3c and d. The comparison between the quantum mutual information and classical correlation reveals that at the gap, where the quantum discord is minimized, the quantum mutual information and classical correlation are maximized. This means that at the gap mentioned, the modes totally become separable, and there is no quantumness between them. To discuss more technically, one may consider the definition of the Gaussian quantum discord, one-way classical correlation, and quantum mutual information as D(ρ_AB_) = h(b)–h (ν_-_)–h (ν_+_) + h (τ + η), C(ρ_AB_) = h(a)—h (τ + η), and I(ρ_AB_) = h(a)–h (ν_–_)–h (ν_+_)^5–7^. The common point between D(ρ_AB_) and I(ρ_AB_) is –[h (ν_–_) + h (ν_+_)], which relates to the von Neumann entropy of the CM; however, the difference between them contributes to the oscillator’s von Neumann entropy (h(b) and h(a)), and h (τ + η) which just appears in D(ρ_AB_). In Fact, the term h (τ + η) comes from the classical correlation effect, which minimizes the classical correlation to maximize the quantum correlation. Using the mentioned points and comparing Fig. 3a with c, one can find that the quantum discord is maximized where the quantum mutual information is minimized. The logarithmic inset graphs in Fig. 3c (Fig. 3c1 and c2) shows clearly the points.

The main difference between the D(ρ_AB_) and C(ρ_AB_) is the last term in the expressions h (τ + η), denoting the measurement effects on the second oscillators. The classical correlation is the maximum information about one subsystem, which depends on the measurement performed on the other subsystem. The result of the classical correlation is demonstrated in Fig. 3d. Also, some inset figures in the logarithmic scale are attached to clearly demonstrate the classical correlation as the function of circuit nonlinearity and frequency. This figure also shows that the classical correlation is maximized at the gap where the quantum discord disappeared. This effect contributes to the influence of the first oscillator’s von Neumann entropy. The same effect could be found in the quantum mutual information graph.

**Figure 4:**
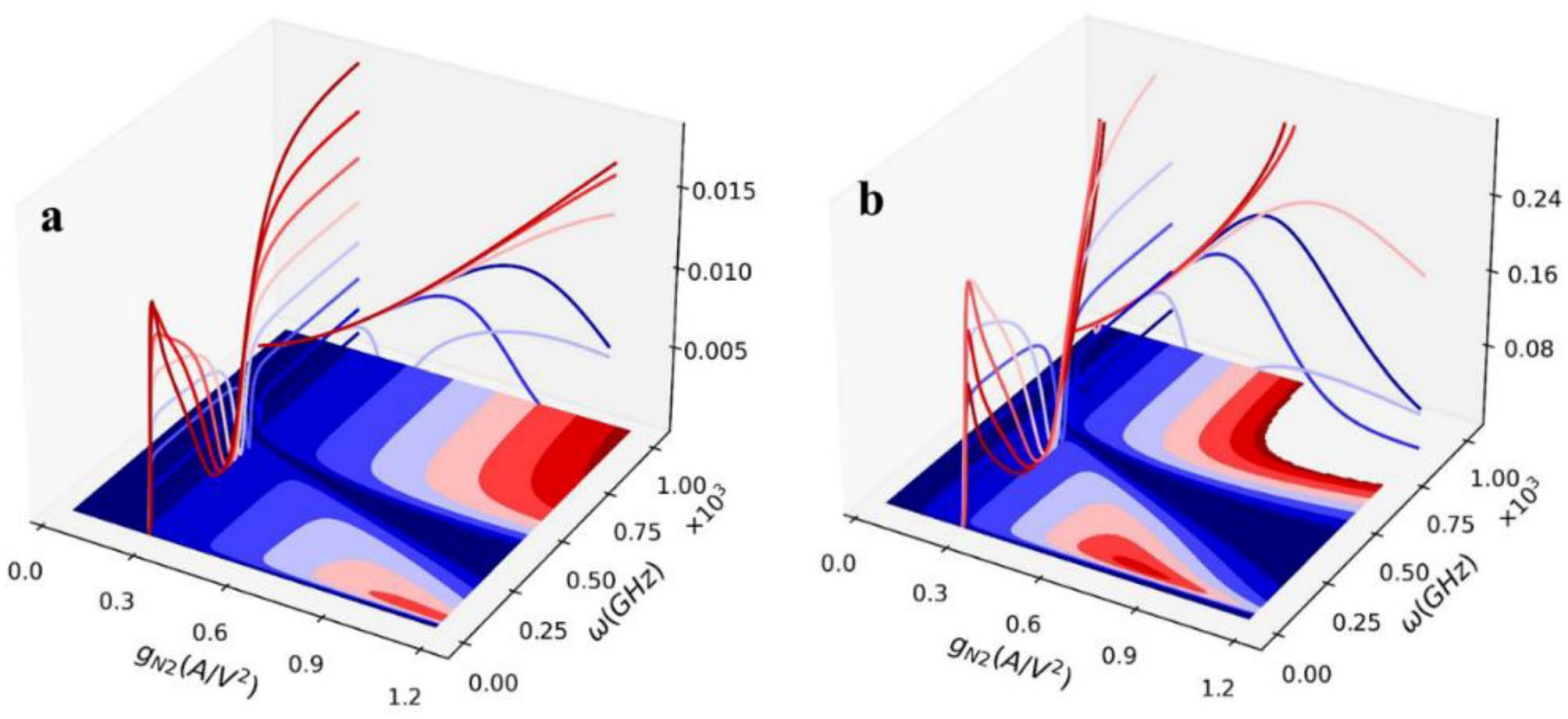
Quantum discord vs. angular frequency (GHz) and nonlinear factor g_N2_ (A/V^2^). (**a**) C_2_ = 0.5 pF, T = 4.2 K, (**b**) T = 1.2 K; C_2_ = 0.5 pF.

It mentioned that this study calculates the Gaussian quantum A discord, meaning that the measurement is performed on the second oscillator. As a result, if one wants to enhance the quantum A discord, it is necessary to minimize the thermally excited photons in the second oscillator. Thus, if one puts most of the thermal photons on the first oscillator (unmeasured subsystem) and decreases the thermal photons in the second oscillator, this enhances the Gaussian quantum discord. In the latter design, C_2_ = 1.0 pF, by which the first and second oscillator’s mean thermally excited photons for a typical frequency were, respectively, n_th1_ = 11.74, n_th2_ = 13.01; also, the mean photon number (phase cross-correlation) for two coupled oscillators was around n_12_ ≡ < δa_1_δa_2_ >  = 2.63e–4. Since C_2_ is changed to 0.5 pF, the mean number of the thermally excited photons in the same frequency becomes n_th1_ = 11.73, n_th2_ = 9.38, and n_12_ = 9.95e–4. The estimated results show that the quantum A discord should be increased. The simulation result is shown in Fig. 4a as a 3D graph. It is shown that the quantum discord amplitude is increased. This is because the second oscillator’s thermally excited photons are decreased. Using illustrated 3D figure, one can get more information about the Gaussian quantum discord since the change of the quantifier as the 1D curves in the x-axis and y-axis are annexed in the figures. The other graph in Fig. 4b shows the operating temperature effect on the quantum A discord. We suppose that if InP HEMT could operate at 1.2 K, what happens on the quantum A discord? Fig. 3b shows a significant enhancement in quantum discord amplitude. This directly contributes to the level of the thermally excited noise generations, which dramatically decreases as the temperature drop from 4.2 to 1.2 K. The other interesting point is that by reducing the temperature to around 1.2 K, at lower g_N2_ the quantum discord amplitude is increased. In the following, we will study that increasing g_N2_ can dramatically change the InP HEMT DC characterization, by which, for instance, the power dissipation can be severely increased. In other words, manipulating g_N2_ to enhance the quantum discord adds a crucial trade-off to RF circuit engineering.

**Figure 5:**
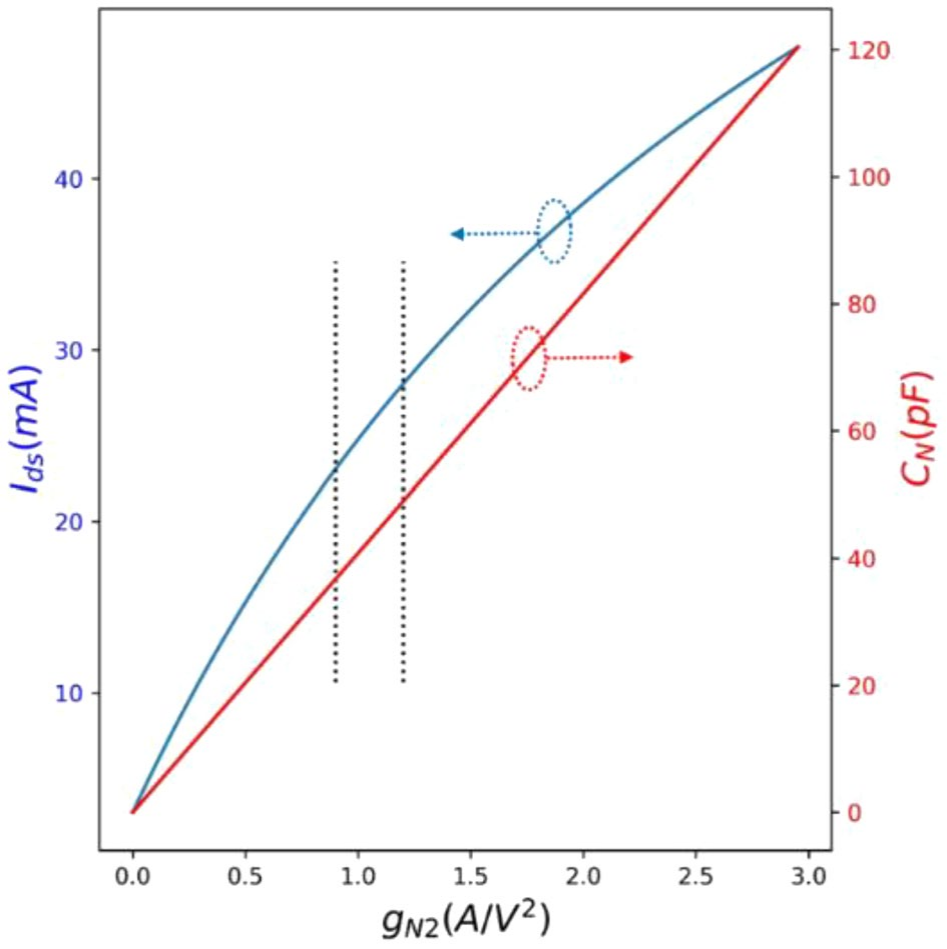
InP HEMT DC characterization: I_ds_ (mA) vs. g_N2_ (A/V^2^), Nonlinear capacitance C_N_ (pF) vs. g_N2_ (A/V^2^).

So far, the study shows that by engineering some parameters related to the internal circuit of the InP HEMT specially focused on the nonlinearity factors, it was possible to enhance the quantum correlation between the two-mode Gaussian states. Initially, it was theoretically shown that the states generated by the InP HEMT nonlinear circuit became mixed. The results showed that the quantum discord is increased for g_N2_ between 0.8 and 1.2 A/V^2^, meaning that the quantum correlation may be created between the states. Finally, arising a trade-off in the design was discussed, which means that one cannot increase g_N2_ to any value to get the desired quantum correlation. It is because the nonlinearity factors can dramatically change the modified circuit performance (DC and AC characteristics). In other words, by changing the nonlinearity factors such as g_m2_ and g_m3_, for example, the DC characteristics of the InP HEMT can be changed. The mentioned variations contribute to the circuit’s components changing, such as C_N_ and g_N2_. To show this point, the circuit illustrated in Fig. 2 is simulated in PySpice (Jupyter Lab) using the data from Table 1. We simulated the internal circuit of InP HEMT, and the related DC characterization is shown in Fig. 5. The left curve as a blue graph shows the InP HEMT drain-source current (I_ds_) as the function of g_N2_, whereas the right graph (red) displays the C_N_ as the function of g_N2_. In the region shown between the dotted line, where the g_N2_ is changed between 0.8 and 1.2, I_ds_ is increased to ~ 30 mA, and C_N_ is reached around 50 pF. It is better to note that I_ds_ is altered in the range of (0.5–2.3) mA for sub-mW cryogenic applications^18^. The mentioned trade-off becomes crucially created, and the designers should especially care about this point. In other words, increasing the current to 30 mA may generate the quantum correlation between states. Still, it forces the system to dissipate energy dramatically, which is crucial in cryogenic applications.

As an essential conclusion of this work, it can be mentioned that InP HEMT nonlinearity has the potential to partially generate the quantum correlation between modes of the oscillators coupled through the transistor. Some critical factors play a central role, such as the nonlinear capacitor arising due to the nonlinearity C_N_ and the nonlinearity factor g_N2_ affected by g_m2_ and g_m3_. These parameters give engineers a reasonable degree of freedom to effectively design a cryogenic circuit containing InP HEMT by which the generation of the quantum correlation between modes at 4.2 becomes possible. However, the generation of entangled microwave photons by InP HEMT operating at 4.2 K seems impossible, at least with the recent technology.

## Conclusions

This study investigated the quantum correlation of the microwave two-mode squeezed thermal state generated by the nonlinearity of InP HEMT. For this reason, the nonlinear circuit related to InP HEMT was analyzed using quantum theory, and the contributed dynamics equation of the motion of the circuit was theoretically derived. In this work, we intensely concentrated on the nonlinear Hamiltonian by which it was theoretically shown that the two-mode squeezed thermal state was generated. In addition, for the circuit discussed, it was theoretically proved that the circuit just generated the mixed state, and there was no pure state. Because of the facts mentioned, the study focused on quantum discord rather than quantum entanglement. To completely know about the quantum discord, other quantities such as quantum mutual information, classical correlation, and the smaller Symplectic eigenvalue were analyzed.

Some engineering was carried out on the nonlinear circuit to get the desired results, and some critical parameters, such as g_m2_ and g_m3,_ were manipulated to enhance the quantum correlation between the generated modes. As an important conclusion, the simulation result showed that although it might be possible to improve the quantum correlation between modes, attaining quantum discord greater than unity seems challenging when InP HEMT operated at 4.2 K.

## Supplementary Information

Supplementary Information.

## Data Availability

Data are available from the corresponding author (Ahmad Salmanogli) upon reasonable request.
