# Supplementary material for: Quantum correlation of microwave two-mode squeezed state generated by nonlinearity of InP HEMT
Source: Sci Rep. 2023 Jul 17;13:11528. doi: 10.1038/s41598-023-37739-0 (PMC10352244; doi:10.1038/s41598-023-37739-0)
Supplement: Supplementary file 1 — Supplementary Information. [file 41598_2023_37739_MOESM1_ESM.docx]

Appendix A:

The linear Hamiltonian of the system is given by:

 (A1)

where C_q1_, C_q2_, C_q1q2_, L_p2_, g_12_, g_22_, V_q1_, V_q2_, and I_p2_ are constants and are defined as:

 (A2)

 (A3)

$\begin{matrix} \gamma_{a11}=-4ig_{N11}\mathrm{Re}\left\{A_{2}\right\} \\ \\ \gamma_{a12}=-4ig_{N11}\mathrm{Re}\left\{A_{1}\right\}-4g_{N41}\mathrm{Re}\left\{A_{2}\right\}+2g_{N61}\left\{A_{1}\right\}^{*} \\ \\ \gamma_{a13}=4g_{N61}\mathrm{Re}\left\{A_{2}\right\} \\ \\ \gamma_{a21}=-4g_{N11}\mathrm{Im}\left\{A_{2}\right\}+2ig_{N21}\mathrm{Re}\left\{A_{2}\right\}+4g_{N41}\mathrm{Re}\left\{A_{2}\right\}-2g_{N61}\mathrm{Re}\left\{A_{1}\right\} \\ \\ \gamma_{a22}=-2g_{N21}\mathrm{Im}\left\{A_{1}\right\}+12ig_{N31}\mathrm{Re}\left\{A_{2}\right\}+4ig_{N41}\mathrm{Im}\left\{A_{1}\right\}-4g_{N51}\mathrm{Re}\left\{A_{2}\right\}+4ig_{N51}\mathrm{Im}\left\{A_{2}\right\} \\ \\ \gamma_{a22}=4g_{N51}\mathrm{Re}\left\{A_{2}\right\} \\ \\ \gamma_{a23}=-2ig_{N61}\mathrm{Im}\left\{A_{1}\right\} \end{matrix}$ (A4)

 (A5)

Using the Hamiltonian expressed in A1 and the related dynamic equation of motion, DC points (constant points) of the circuit can be calculated. Therefore, the steady state equations become:

 (A6)

Using the standard approach to solve the equations, the final form of the variables become:

 (A7)

where A_1_ = A_1R_+iA_1j_ and A_2_ = A_2R_+iA_2j_. Using Eq. A7 the steady state point of the circuit, which is related to the thermal noise and bias point of the circuit, is calculated.
